# Supplementary material for: Comparison of mean platelet volume (MPV) and red blood cell distribution width (RDW) between psoriasis patients and controls: A systematic review and meta-analysis
Source: PLoS One. 2022 Feb 25;17(2):e0264504. doi: 10.1371/journal.pone.0264504 (PMC8880915; doi:10.1371/journal.pone.0264504)
Supplement: S1 Table — (PDF) [file pone.0264504.s002.pdf]

S1 Table. Literature searching strategy.

**PubMed**		
#1	Search "Psoriasis"[Mesh]	39345
#2	Search ((((((psoriasis[Title/Abstract]) OR Psoriases[Title/Abstract]) OR (Pustulosis of Palms[Title/Abstract] AND Soles[Title/Abstract])) OR Pustulosis Palmaris et Plantaris[Title/Abstract]) OR Palmoplantaris Pustulosis[Title/Abstract]) OR (Pustular Psoriasis of Palms[Title/Abstract] AND Soles[Title/Abstract])) OR PSO[Title/Abstract]	41724
#3	Search (((((((((Arthritis, Psoriatic[Title/Abstract]) OR Psoriasis, Arthritic[Title/Abstract]) OR Arthritic Psoriasis[Title/Abstract]) OR Psoriatic Arthritis[Title/Abstract]) OR Psoriasis Arthropathica[Title/Abstract]) OR Psoriatic Arthropathy[Title/Abstract]) OR Arthropathies, Psoriatic[Title/Abstract]) OR Arthropathy, Psoriatic[Title/Abstract]) OR Psoriatic Arthropathies[Title/Abstract]) OR PSA[Title/Abstract]	40756
#4	#1 OR #2 OR #3	85446
#5	Search "Mean Platelet Volume"[Mesh]	1125
#6	Search ((((((((Mean platelet volume[Title/Abstract]) OR Mean Platelet Volumes[Title/Abstract]) OR Platelet Volume, Mean[Title/Abstract]) OR Platelet Volumes, Mean[Title/Abstract]) OR Volume, Mean Platelet[Title/Abstract]) OR Volumes, Mean Platelet[Title/Abstract]) OR MPV[Title/Abstract]) OR platelet volume[Title/Abstract]) OR platelet volumes[Title/Abstract]	6515
#7	#5 OR #6	6589
#8	Search "Erythrocyte Indices"[Mesh]	5346
#9	Search ((((((Erythrocyte Indices[Title/Abstract]) OR Red Cell Distribution Width[Title/Abstract]) OR red blood cell distribution width[Title/Abstract]) OR Red Cell Volume Distribution Width[Title/Abstract]) OR Red Cell Distributed Width[Title/Abstract]) OR Red Cells Distribution Width[Title/Abstract]) OR RDW[Title/Abstract]	2786
#10	#8 OR #9	6918
#11	#7 OR #10	12922
#12	#4 AND #11	40
**Embase**		
#1	'psoriasis'/exp	89666
#2	'psoriasis':ab,ti OR 'psoriases':ab,ti OR 'pustulosis of palms and soles':ab,ti OR 'pustulosis palmaris et plantaris':ab,ti OR 'palmoplantaris pustulosis':ab,ti OR 'pustular psoriasis of palms and soles':ab,ti OR 'pso':ab,ti	63031
#3	'arthritis, psoriatic':ab,ti OR 'psoriasis, arthritic':ab,ti OR 'arthritic psoriasis':ab,ti OR 'psoriatic arthritis':ab,ti OR 'psoriasis arthropathica':ab,ti OR 'psoriatic arthropathy':ab,ti OR 'arthropathies, psoriatic':ab,ti OR 'arthropathy, psoriatic':ab,ti OR 'psoriatic arthropathies':ab,ti OR 'psa':ab,ti	76009

#4	#1 OR #2 OR #3	156385
#5	'mean platelet volume'/exp	1550
#6	'mean platelet volume':ab,ti OR 'mean platelet volumes':ab,ti OR 'platelet volume, mean':ab,ti OR 'platelet volumes, mean':ab,ti OR 'volume, mean platelet':ab,ti OR 'volumes, mean platelet':ab,ti OR 'mpv':ab,ti OR 'platelet volume':ab,ti OR 'platelet volumes':ab,ti	6943
#7	#5 OR #6	7285
#8	'red cell distribution width'/exp	408
#9	'red cell distribution width':ab,ti OR 'red blood cell distribution width':ab,ti OR 'red cell volume distribution width':ab,ti OR 'red cell distributed width':ab,ti OR 'red cells distribution width':ab,ti OR 'rdw':ab,ti OR 'erythrocyte indices':ab,ti	4608
#10	#8 OR #9	4697
#11	#7 OR #10	11395
#12	#4 AND #11	55
**Cochrane library**		
#1	MeSH descriptor: [Psoriasis] explode all trees	3206
#2	(psoriasis):ti,ab,kw OR (Psoriases):ti,ab,kw OR (Pustulosis of Palms and Soles):ti,ab,kw OR (Pustulosis Palmaris et Plantaris):ti,ab,kw OR (Palmoplantaris Pustulosis):ti,ab,kw OR (Pustular Psoriasis of Palms and Soles):ti,ab,kw OR (PSO):ti,ab,kw	7582
#3	(Arthritis, Psoriatic):ti,ab,kw OR (Psoriasis, Arthritic):ti,ab,kw OR (Arthritic Psoriasis):ti,ab,kw OR (Psoriatic Arthritis):ti,ab,kw OR (Psoriasis Arthropathica):ti,ab,kw OR (Psoriatic Arthropathy):ti,ab,kw OR (Arthropathies, Psoriatic):ti,ab,kw OR (Arthropathy, Psoriatic):ti,ab,kw OR (Psoriatic Arthropathies):ti,ab,kw OR (PSA):ti,ab,kw	6950
#4	MeSH descriptor: [Mean Platelet Volume] explode all trees	8
#5	(Mean Platelet Volume):ti,ab,kw OR (Mean Platelet Volumes):ti,ab,kw OR (Platelet Volume, Mean):ti,ab,kw OR (Platelet Volumes, Mean):ti,ab,kw OR (Volume, Mean Platelet):ti,ab,kw OR (Volumes, Mean Platelet):ti,ab,kw OR (MPV):ti,ab,kw OR (platelet volume):ti,ab,kw OR (platelet volumes):ti,ab,kw	1602
#6	MeSH descriptor: [Erythrocyte Indices] explode all trees	169
#7	(Indices, Erythrocyte):ti,ab,kw OR (Red Cell Indices):ti,ab,kw OR (Red Cell Index):ti,ab,kw OR (Index, Red Cell):ti,ab,kw OR (Indices, Red Cell):ti,ab,kw OR (Red Cell Indexes):ti,ab,kw OR (Indexes, Red Cell):ti,ab,kw OR (Erythrocyte Index):ti,ab,kw OR (Index, Erythrocyte):ti,ab,kw OR (Erythrocyte Indexes):ti,ab,kw OR (Indexes, Erythrocyte):ti,ab,kw OR (Red Cell Distribution Width):ti,ab,kw OR (red blood cell distribution width):ti,ab,kw OR (Red Cell Volume Distribution Width):ti,ab,kw OR (Red Cell Distributed Width):ti,ab,kw OR (Red Cells Distribution Width):ti,ab,kw OR (RDW):ti,ab,kw	2287
#8	#1 OR #2 OR #3	13471
#9	#4 OR #5 OR #6 OR #7	3847
#10	#8 AND #9	39
