# Supplementary material for: Comparison of mean platelet volume (MPV) and red blood cell distribution width (RDW) between psoriasis patients and controls: A systematic review and meta-analysis
Source: PLoS One. 2022 Feb 25;17(2):e0264504. doi: 10.1371/journal.pone.0264504 (PMC8880915; doi:10.1371/journal.pone.0264504)
Supplement: Attachment — Submitted filename: Response to Reviewers.docx [file pone.0264504.s003.docx]

**Response to Reviewers**

Thanks for your thoughtful and constructive comments. We have learned a lot from these comments, and addressed all of questions accordingly. Please find the revised texts highlighted in the revised manuscript, and the point-by-point responses as follows:

Journal Requirements:

When submitting your revision, we need you to address these additional requirements.

1. Please ensure that your manuscript meets PLOS ONE's style requirements, including those for file naming. The PLOS ONE style templates can be found at 
https://journals.plos.org/plosone/s/file?id=wjVg/PLOSOne_formatting_sample_main_body.pdf and 
<https://journals.plos.org/plosone/s/file?id=ba62/PLOSOne_formatting_sample_title_authors_affiliations.pdf>

**Response:** We have checked the manuscript carefully and ensured that our manuscript meets PLOS ONE's style requirements, including file naming.

2. We note that you have stated that you will provide repository information for your data at acceptance. Should your manuscript be accepted for publication, we will hold it until you provide the relevant accession numbers or DOIs necessary to access your data. If you wish to make changes to your Data Availability statement, please describe these changes in your cover letter and we will update your Data Availability statement to reflect the information you provide.

**Response:** We have changed data availability statement to “all relevant data are within the manuscript and its Supporting Information files”, since there is no additional data for this manuscript.

3. Please amend either the abstract on the online submission form (via Edit Submission) or the abstract in the manuscript so that they are identical.

**Response:** We have ensured that abstract on the online submission and the abstract in the manuscript are identical.

4. Please include captions for your Supporting Information files at the end of your manuscript, and update any in-text citations to match accordingly. Please see our Supporting Information guidelines for more information: http://journals.plos.org/plosone/s/supporting-information. 
**Response:** We have included captions for our Supporting Information files at the end of our manuscript, and updated the in-text citation to match accordingly.

Reviewers' comments:

**Reviewer #1:** This is a meta-analysis of RDW and MPV as biomarkers and predictors of disease severity by Jiang et al.

1. Which drugs affecting hematologic diseases were excluded? Was biotin also excluded?

**Response:** Thanks for your comment. We have excluded the antiplatelet drugs and nonsteroid anti-inflammatory drugs but not biotin, because the measurement of MPV and RDW is independent of biotin in blood (certain laboratory results influenced by biotin include troponin, thyroid and other hormones and vitamin D level) (https://labtestsonline.org/articles/biotin-affects-some-blood-test-results).

2. Was there a reason why male or female only studies were excluded? While there are potential gender differences in values, you could also run a subset analysis based on gender. Please include reasoning.

**Response:** Thanks for your important comment. Indeed, only one monosexual study was excluded based on this criterion. The reason for establishing this standard is that all enrolled studies focused on both the male and female except one study by Ozlem Karabudak in 2008. As you mentioned, subset analysis could be used to diminish the effect of gender, however, only two included studies have provided the MPV value stratified by gender. Consequently, data is insufficient to get credible results for the subset analysis of gender. In order to avoid bias caused by gender, we have excluded this study by exclusive standard.

3. In limitations, this reviewer suggests adding that it's possible that even aggregated the included studies are not sufficient to detect if there is a difference.

**Response:** We have added “it's possible that even aggregated the included studies are not sufficient to detect if there is a difference” in limitations.

**Minor comments:**
1. Suggest adding a transition sentence between promising biomarkers and hematologic parameters to improve flow

**Response:** Thanks for your suggestion. We have added a transition sentence between promising biomarkers and hematologic parameters in introduction section to improve flow.

2. There are spelling/grammar/word errors throughout the paper and highlighting several here:
Introduction- change "The diagnose the psoriasis" to "The diagnosis of psoriasis"

**Response:** We have changed "The diagnose the psoriasis" to "The diagnosis of psoriasis"

in introduction section.

Exclusion standards- change retreated to retracted

**Response:** We have changed “retreated” to “retracted” in exclusion standards.

Results-11 and 12 should be spelled out

**Response:** We have spelled out 11 and 12 in results.

Figure 1- psoriasis is misspelled

**Response:** We have corrected the spelling mistake in figure 1.

**Meanwhile, we have our manuscript polished by a native English speaker to make it more readable.**

**Reviewer #2:** The present meta-analysis aimed at investigating the association of MPV and RDW with diagnosis and disease severity of psoriasis by extracting data from thirteen studies appropriately chosen according to certain criteria.
The manuscript provides robust statistical analysis on the extracted data to assess the association of MPV and RDW with psoriasis, the heterogeneity among studies and the entity of the publication bias, as well as sensitivity and meta-regression analysis. Nevertheless, more details are needed to clarify the biological aspects related to MPV and RDW involvement in the pathogenesis of psoriasis.

1. In the Discussion Section about MPV, authors mention findings from other studies that demonstrate elevation of this parameter in psoriasis patients, suggesting the involvement of increased platelet activation in the pathogenesis of psoriasis. Authors should give a deeper explanation on how MPV can be affected by the clinical-molecular alterations underlying the disease onset and progression, outlining possible mechanisms, pathways or molecular factors related (for instance, recent articles/reviews concerning the role of angiogenesis, cell proliferation, inflammation could be employed for a better introduction of the topic).
**Response:** Thanks for your constructive comment! We have cited more related literation to elaborate MPV-involved possible mechanisms underlying the disease onset and progression. Platelets synthesize, store, and secret inflammatory factors to induce inflammatory response. At the same time, they express P-selectin, CD40 ligand, Toll-like receptors, and integrins on their surface for the recruitment of polymorphonuclear neutrophils, cell adhesion and migration, as well as interaction with immune cells in the onset and progression of psoriasis.

2. Previous literature suggested RDW as a biomarker of cardiovascular disease susceptibility and inflammation levels in rheumatoid arthritis (RA) and ankylosing spongdylitis (AS). In the Discussion section, authors do not explore the connection between the variation of volume and size of red blood cells (measured by RDW) and the pathological processes underlying the development of psoriasis. Therefore, authors should explain how this parameter could be involved in the pathogenesis of the disease, justifying why clinicians should consider RDW informative in psoriasis patients especially regarding its predictive role in response to treatment.
**Response:** Thanks for your important comment. We pretty agree with your point of view. However, the erythroid disturbance in psoriasis patients has been hardly reported. A few studies reported that increased inflammatory cytokines in the skin and peripheral blood of psoriasis patients influenced erythropoiesis and elevated the value of RDW. We have cited and discussed these articles in the discussion section (page 17-18). Moreover, further studies are needed to have a more in-depth sight.

3. This meta-analysis demonstrates a correlation of MPV and RDW with the presence of psoriasis rather than with disease severity. Authors should implement the conclusion suggesting how this finding could help clinicians with the disease management, selection of therapies and patient follow-up, since they purpose these parameters as promising predictive and diagnostic biomarkers of psoriasis.
**Response:** Thanks for your comment. The current study could only demonstrate the correlation of MPV and RDW with the presence of psoriasis. Further analysis such as the disease severity and long-term prognosis is limited by unavailable data currently. Thus, we could only point out that MPV and RDW contribute to the early diagnosis of psoriasis for clinicians. As we know, patients could benefit a lot from early diagnosis and treatment. Besides, several studies have showed that the induction of these two indicators possibly provide reference significance for the judgement of treatment efficacy in the short term, however the comprehensive analysis of several hematological parameters are needed for efficacy evaluation in the long term. We have further elucidated the above opinion in the discussion section.

4. References must have the same format even if they come from different databases to allow users easier searching on the source of information mentioned in the manuscript. Therefore, authors should review the bibliography inserting some missing information for example the Digital Object Identifier (doi) and the source of the cited article in all references.

**Response:** We have reviewed the bibliography and inserted some missing information including doi and the source of the cited article in all references except for Ref. 26, 46 and 50 without available doi.
